# Validation of Contamination Control in Rapid Transfer Port Chambers for Pharmaceutical Manufacturing Processes

**DOI:** 10.3390/ijerph13111129

**Published:** 2016-11-12

**Authors:** Shih-Cheng Hu, Angus Shiue, Han-Yang Liu, Rong-Ben Chiu

**Affiliations:** 1 Department of Energy and Refrigerating Air-Conditioning Engineering, National Taipei University of Technology, Taipei 10608, Taiwan; schu.ntut@gmail.com; 2 Air System Enterprise Co., Ltd., Taoyuan 326, Taiwan; asecoltd@ms27.hinet.net (H.-Y.L.); asentco@ms63.hinet.net (R.-B.C.)

**Keywords:** rapid transfer port, contamination, cleanroom, airflow pattern, particle image velocimetry

## Abstract

There is worldwide concern with regard to the adverse effects of drug usage. However, contaminants can gain entry into a drug manufacturing process stream from several sources such as personnel, poor facility design, incoming ventilation air, machinery and other equipment for production, etc. In this validation study, we aimed to determine the impact and evaluate the contamination control in the preparation areas of the rapid transfer port (RTP) chamber during the pharmaceutical manufacturing processes. The RTP chamber is normally tested for airflow velocity, particle counts, pressure decay of leakage, and sterility. The air flow balance of the RTP chamber is affected by the airflow quantity and the height above the platform. It is relatively easy to evaluate the RTP chamber′s leakage by the pressure decay, where the system is charged with the air, closed, and the decay of pressure is measured by the time period. We conducted the determination of a vaporized H_2_O_2_ of a sufficient concentration to complete decontamination. The performance of the RTP chamber will improve safety and can be completely tested at an ISO Class 5 environment.

## 1. Introduction

The pharmaceutical material transport into the Grade A (equivalent to ISO 5 (ISO 14644-1) [1]) filling environments is one of the most common for the causes of the aseptic processing deterioration [2]. The design arrangements of the conventional filling facility have placed it in a Grade B environment. Process operations are required while sterile materials are delivered from a Grade B environment to the Grade A filling area. Contamination is a key factor in risk analysis of classical aseptic processing areas. Open processes, which need direct operator intrusion, are considered to be a greater risk for contamination than closed processes. The execution of the isolator technology has noticeably decreased the impact of the contaminated surrounding environment upon the vital zone of the aseptic processing. Although the isolator is properly validated and operated which can decrease risk from an environmental contamination to a level approaching zero, the transfer of materials in and out of an isolator is the most probable reason for the loss of the separated enclosures environmental integrity. This is often accomplished by correspondence the process and the activity of people and materials, by utilizing risk assessment tool such as Hazard Analysis and Critical Control Points [3] or Failure Modes and Effects Analysis [4]. The safer the transfer system, the more the external environment becomes less threatening, and therefore it results in a more efficient risk management. The Parenteral Drug Association (PDA) Industry Aseptic Processing Survey 2001 identified the material transfer failures as follows [5]:

- Personnel contamination
- Non-routine activity
- Aseptic assembly
- Human error
- Mechanical failure
- Airborne contaminants
- Improper sanitization: Surface contaminants
- Material transfers: Failure of HEPA (High Efficiency Particulate Air Filter) (0.2 μm filter)
- Improper sterilization

The transfer technology is a major part of the manufacturing pharmaceutical isolator (Class III Biological Safety Cabinet) systems and should be selected according to the provided protection level. Hence, the available transfer technologies are air-lock, rapid transfer ports (RTP) chamber, and laminar-airflow interface. The RTP chamber is generally utilized for physical separation [1,6]. Firstly, it can be built into a wall or incorporated in the isolator barrier system with a transfer chamber to supply a secure and simple method for removing articles, materials, supplies and waste without rupturing the containment [7,8]. Secondly, it can be fabricated in various sizes, fastened to connect necessities for choosing agent storage, facilitated to ensure the chain of delivery, and for describing procedure. Thirdly, it is designed to transfer pharmaceutical material, and therefore continuous airflow within the chamber is needed to decrease the concentration of particle contamination [9] to confirm all biological safety standards. The resulting exhaust gas is passed through a designed filtering system to prevent particle contamination from the internal to the ambient environment or contrariwise [10,11,12,13,14]. The RTP chambers were designed to increase experimental safety by prohibiting this contamination, decreased the possibility of operator error, and lessen the contaminated area. The maintenance of the RTP chamber is critical, especially in assuring the minimization of the leakage (where the leakage of air could lead to the ingress of air from the less-clean surrounding room environment) maintenance of the RTP chamber integrity, of which is most commonly sanitized by hydrogen peroxide vapor.

The aim of the study was to demonstrate that the RTP chamber could be utilized as a containment device for the pharmaceutical manufacturing processes. If validated, the device could be used as a primary barrier in high containment to protect the operators and the environment from accidental exposure to aerosols, which is the part of the risk evaluation of the operation via the human pathogens, but distinct leading is not offered where aerosols are deliberately created [15]. In the absence of a published standard for the RTP chamber testing, a test protocol was developed based on a combination of the test standards that are generally utilized in the high-containment environments. This study also presented four tests to evaluate cross-contamination control in the RTP chambers: (1) a quantitative check of the airflow velocities; (2) a particle count test under normal and enhanced airflow velocities; (3) a pressure decay test of the complete containment system at different initial pressures; (4) vaporized hydrogen peroxide decontamination.

## 2. Materials and Methods

**Figure 1:**
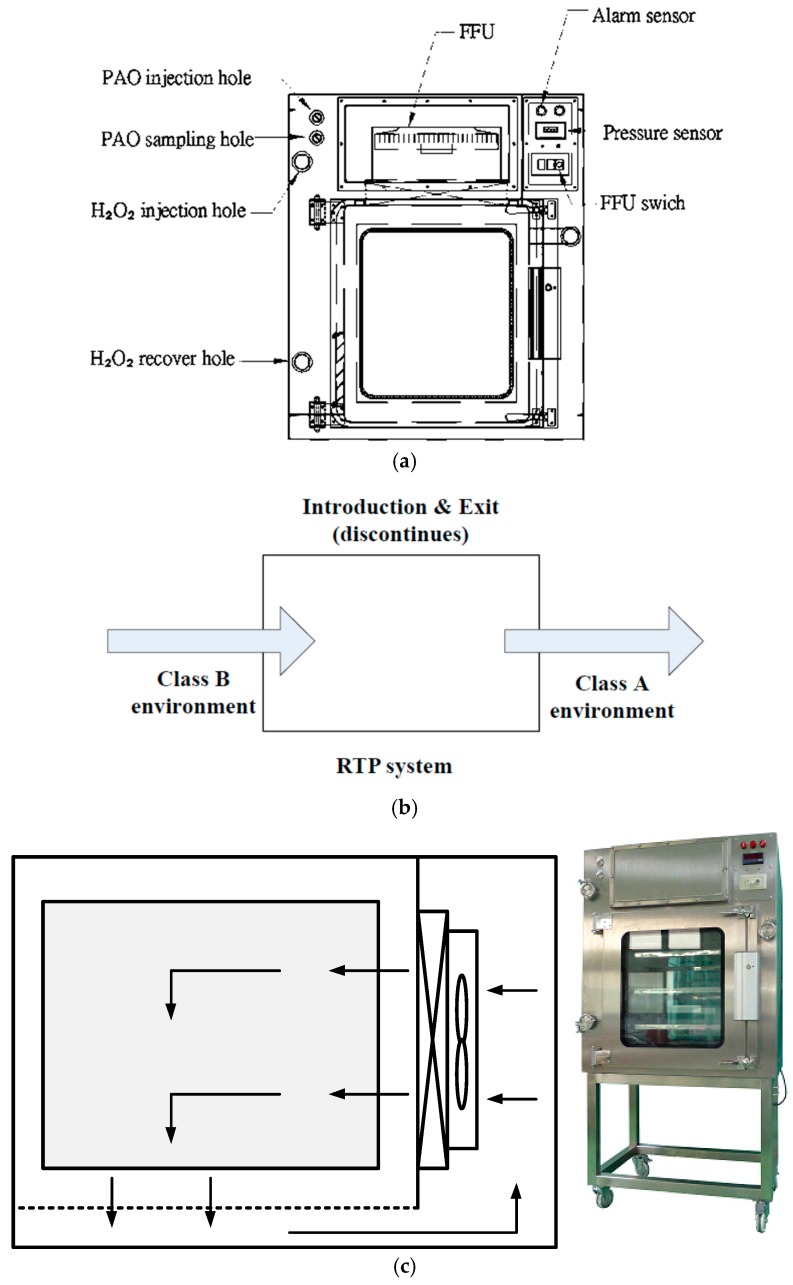
Rapid Transfer Port Chamber (Air System Enterprise Co., Ltd., Taoyuan, Taiwan; Self-Fabricated) (**a**) Schematic; (**b**) Material transfer function; (**c**) Air distribution.

To make a good internal environment of the RTP chamber that is fully isolated from the exterior, variable frequency drive fans were utilized in the air-intake and the air-exhaust system, precisely controlling the filtered air flow and supplying an air-barrier protection. The aerodynamics allows for a one-way flow or airflow turbulence within the RTP chamber, under negative pressure compared to the surrounding environment. Supply and exhaust air can go through the high-efficiency particulate air (HEPA) filters (Efficiency 99.99% on 0.3 micron) to prevent the production of aerosols that could possibly escape into the ambient environment [16]. Following ISO 14644-1 [1], ISO 14644-7 [17], ISO 10648-2 [18], and ASTM (American Society for Testing and Materials) E2930-13 [19] standards, contamination control in the RTP chamber was evaluated. Experiments were carried out using airflow velocity meter (TSI Inc., 9535-L, Cole-Parmer, Taichung, Taiwan), an optical particle counter (Pacific Scientific Model Met One 2100.10, Ashtead Technology, Westhill, UK), a pressure meter (testo480-manual-0563_4800, TESTO, New Taipei, Taiwan) and a vaporized hydrogen peroxide generation detector (self-fabricated) to perform four validation tests (including airflow velocity, particle quantities, pressure decay and vaporized hydrogen peroxide amount) in the RTP chamber (Size: 960 mm (Length) × 600 mm (width) × 1120 mm (height), Air System Enterprise Co., Ltd., Taoyuan, Taiwan) as shown in Figure 1. The RTP chamber assisted the low-contamination inward and outward transfer of the product chambers or system parts into and out of the containment systems. The double door cannot be opened until the chamber is tied and closed in position.

### 2.1. Airflow Velocity Test

**Figure 2:**
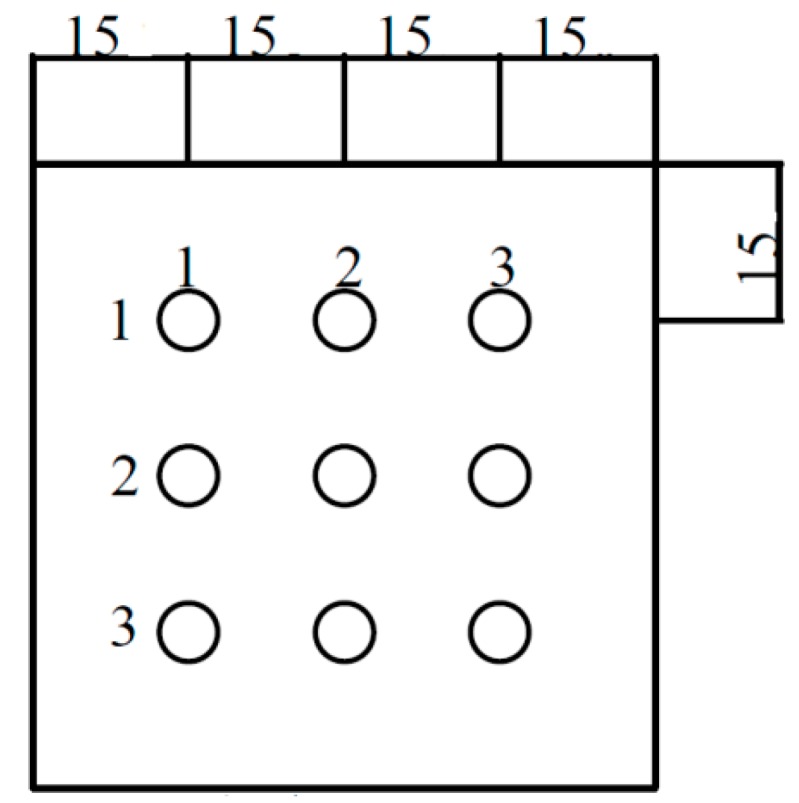
Location of the measuring points during airflow velocity test.

The work zone of the airflow velocities monitoring is described by the airflow at right angles to the supply HEPA filter, often recommended as a grid of equal spaced points on the entrance plane of the airflow. This test measured the removing of the airflow velocity along the RTP work space, 15, 30, 45, 60, and 95 cm above the platform, and is performed at three percentage operating levels (50%, 60% and 75%) on the whole RTP chamber (see Figure 2). Readings at individual locations were obtained and noted on a specified grid via the movable parts and the average for each designated zone is thus calculated [20].

### 2.2. Particle Count Method

**Figure 3:**
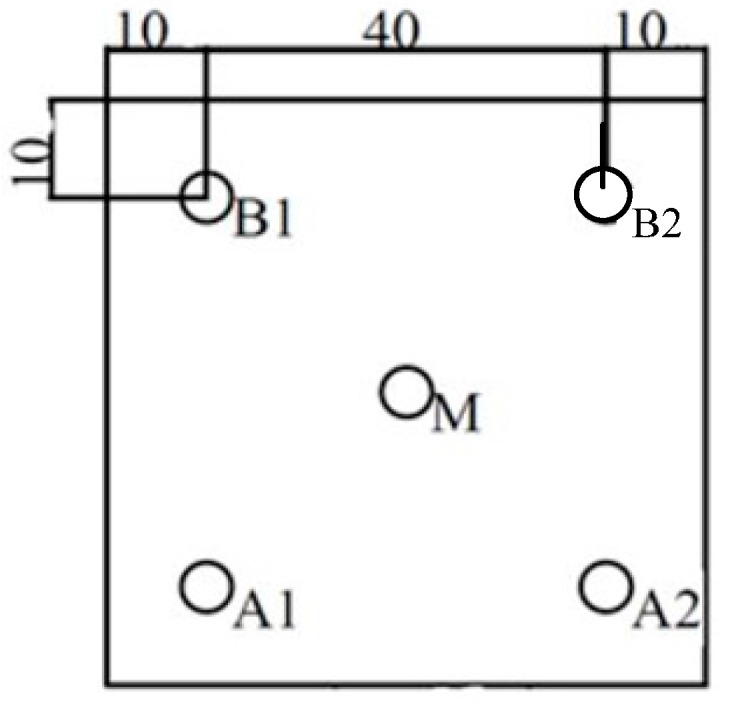
Location of the measuring points during particle measurements.

Particle counting probe should be fixed in an orientation demonstrated to get an important sample and kept to assure aseptic conditions while HEPA filter integrity. The particle count test is executed by particles counting at defined grid locations within the RTP chamber. The sampling points should be equally spaced throughout five locations [21], and at a work height to describe the quality of the air cleanliness as it approaches the RTP chamber as presented in Figure 3.

### 2.3. Pressure Decay Test

First, we sealed the return air and air outlet, and then injected dry air into the RTP chamber to bring the pressure to 15, 30, and 45 Pa. After these steps, we observed the pressure differential by using a pressure meter, and recorded the time required for the pressure dropping to zero Pa [22]. It was noticed what differentiated the ideal gas law: *pV* = *mRT* via the time, given as by Doyle [23].

(1) $$V\frac{dp}{dt}=RT\frac{dm}{dt}$$

where *V*: volume constant, and *T*: temperature constant (as slow leakage from a large system is a quasi-static process). Also, all the leaks inside the entire gaseous nitrogen system can be designed for one large equivalent orifice. For the choked flow, the flowrate through this equivalent orifice is [24].

(2) $$\frac{dm}{dt}(lb_{m}/\sec)=-CAp{\left[\frac{g_{c}}{RT}k{\left(\frac{2}{k+1}\right)}^{\frac{\left(k+1\right)}{\left(k-1\right)}}\right]}^{\frac{1}{2}}$$

where *C*: orifice flow coefficient, *A*: flow area normal to flow direction (in^2^), *p*: upstream pressure (psia), *R*: ideal gas constant (ft-lbf/lbm- R), *g_c_*: unitary conversion factor = 32.2 lbm ft/lbf s^2^, and *k*: ratio of specific heats. The minus sign on the right side of Equation (2) shows that the mass left from the control volume.

Also, make ${\left[g_{c}k{\left(\frac{2}{k+1}\right)}^{\left(k+1)/(k-1\right)}\right]}^{\frac{1}{2}}=S$

Combining Equations (1) and (2) results in:

(3) $$\frac{dp}{dt}=-[\frac{CAS\sqrt{RT}}{V}]p\text{ or }\frac{dp}{dt}+[\frac{CAS\sqrt{RT}}{V}]p=0$$

At the beginning, it was deduced that all the quantities within the parentheses of Equation (3) were constants as for pressure and time. This lead to a simple first order linear differential equation, and the solution is exponential decay:

(4) $$p(t)=p(0)e^{-at}$$

where $a=\frac{CAS\sqrt{RT}}{V}$.

### 2.4. Vaporized Hydrogen Peroxide Fumigation Tests

**Figure 4:**
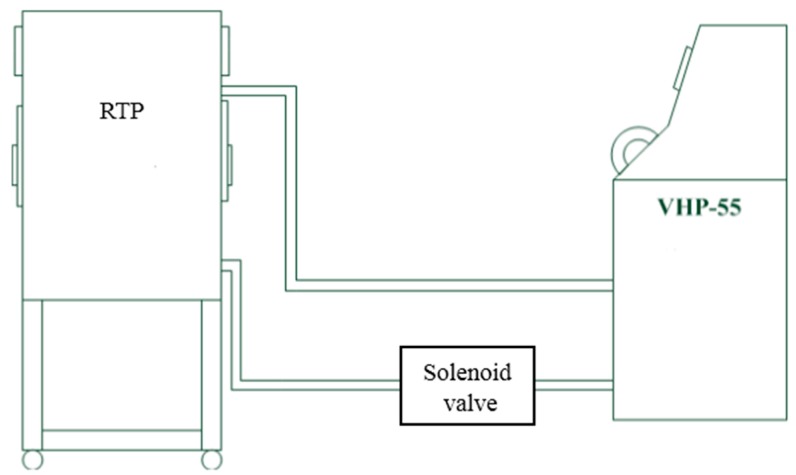
Process Flow for RTP Decontamination.

A self-fabricated VHP generating unit (see Figure 4) uses 35% wt. hydrogen peroxide generating the vapor within the target enclosure to decontaminate the RTP [1]. This generator performs and controls four phases during the sterilization cycle: dehumidification, conditioning, decontamination, and aeration. To initiate the cycle, vaporized hydrogen peroxide is first pumped from the tube to the RTP. During the dehumidification phase, the unit removes moisture from the enclosure to a defined set point prior to the injection of vaporized hydrogen peroxide. The conditioning phase promotes a rapid increase in hydrogen peroxide levels within the enclosure over a relatively short amount of time. The decontamination phase has a reduced injection rate that maintains the vaporized hydrogen peroxide concentration (achieved during conditioning) over an extended period of time. Aeration, in which the vaporous hydrogen peroxide is circulated through HEPA filters and an activated carbon filter for neutralization, is initiated once the programmed decontamination time has been concluded.

## 3. Results and Discussion

### 3.1. Airflow Velocity Test

**Figure 5:**
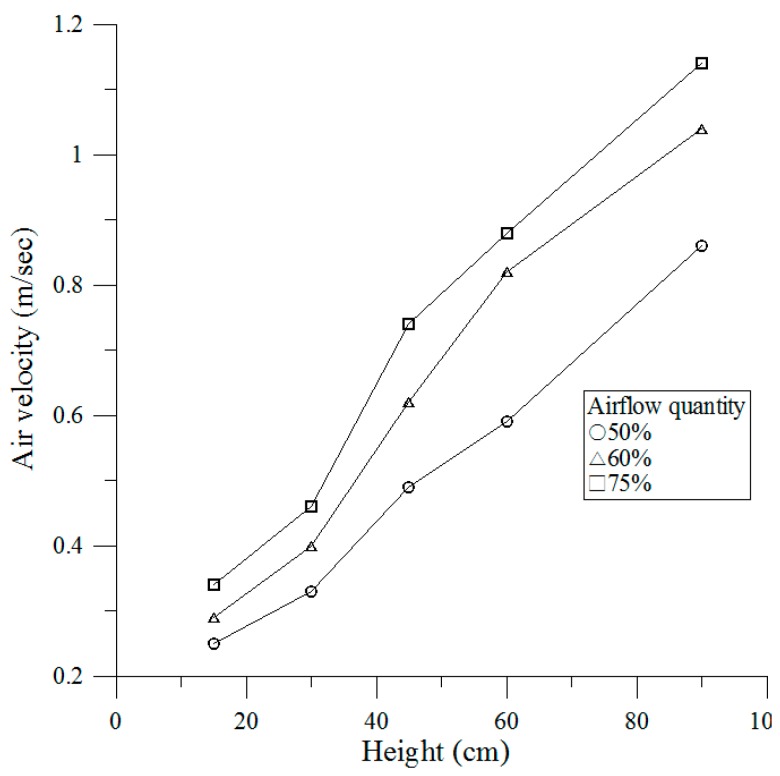
Airflow velocities at different airflow quantity.

The vertical distribution of airflow velocities was determined by measuring 50%, 60% and 75% airflow quantity at varying heights (*h* = 15, 30, 45, 60 and 95 cm). The data shows that the airflow quantity produces a more important character in the airflow velocities. The measuring indicated that the airflow velocities are by the height from the platform substantially depends on 50%, 60% and 75% airflow quantity. The airflow velocities at four tested heights (15, 30, 45, 60 and 95 cm) were linearly related to the tested heights, giving a polynomial relation at the tested heights (15, 30, 45, 60 and 95 cm). Increasing airflow quantity more quickly promotes larger airflow velocities. When relating the effects of airflow velocities to the height using a multiple linear regression technique, we obtained the relationships between the airflow velocity and the height shown as Figure 5. Underneath HEPA filter (95 cm height), average airflow velocity is within ±0.00002 m/s, which are smaller and continued compliance with acceptance testing procedure of ISO 14644-3 C.4 [25].

### 3.2. Particle Count Method

The monitored particle concentration for different points inside the RTP chamber is shown in Table 1. The distribution of the particle concentration attends to be commanded by the airflow velocity and the particle diameters. Table 1 shows the RTP chamber particle concentration via the different airflow velocities. While the airflow velocity is higher, the consistence of the particle concentration is preferable. It presents that strengthening the convection can also modify the consistence of the concentration. All the results presented in Table 1 demonstrate that enlarging the particle diameter can decrease the consistence of the concentration. The dissimilarity of the concentration between different points is obvious for particles with diameter larger than 3 μm. The difference between the different positions can surpass 30% for particles with a diameter smaller than 1 μm in the RTP chamber. The dissimilarity of the concentration at the same point between the particles with different sizes can also surpass 30%. The middle measuring point M is the best cleanliness method/technique due to the use of a centrifugal fan. Particle counts above zero indicate integrity deficits. HEPA filter integrity should be kept to assure aseptic conditions. The cleanliness classification of the RTP chamber work zone (as defined by USP 797 [26]) continued compliance with acceptance testing procedure of ISO class 5 at 0.5 µm and larger particles under operational conditions.

### 3.3. Pressure Decay Tests

**Figure 6:**
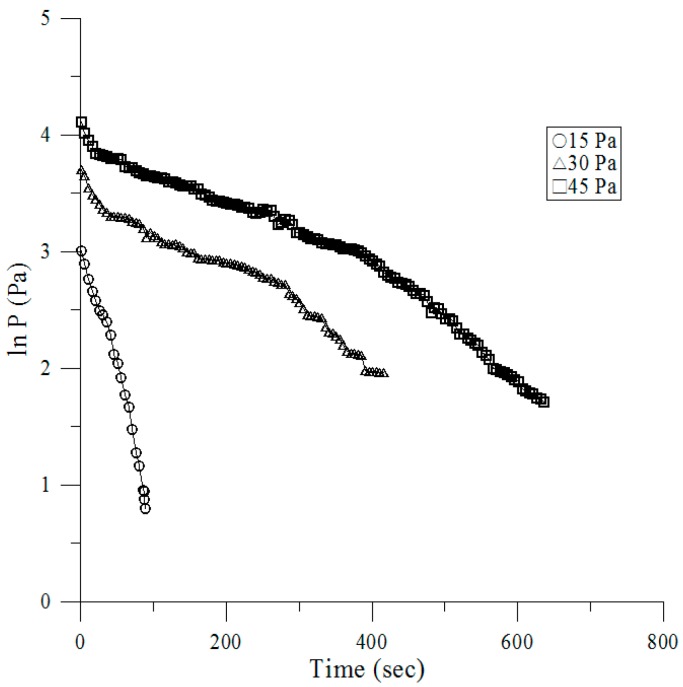
Pressure (*P*) versus time (*t*).

Figure 6 presents pressure versus time for the air at an ambient temperature via 15, 30, and 45 Pa of three different initial pressures. Once the initial pressure value was obtained, the charge valve was closed. Gas leaked through the system from the metering valve, and utilized the passing time, as the pressure decayed under 8 Pa. RTP chamber employed a positive air pressure differential adequate to achieve this full separation continued compliance with acceptance testing procedure of the FDA (Food and Drug Administration) guidelines [27]. The smaller the value of the initial pressure makes it faster than the initial decay rate. The proportional relationship between the natural logarithm of decay time and the pressure is presented in a simple linear formula and the correlation coefficient is over 0.961 for all of the results as shown in Table 2.

### 3.4. Vaporized Hydrogen Peroxide Tests

**Figure 7:**
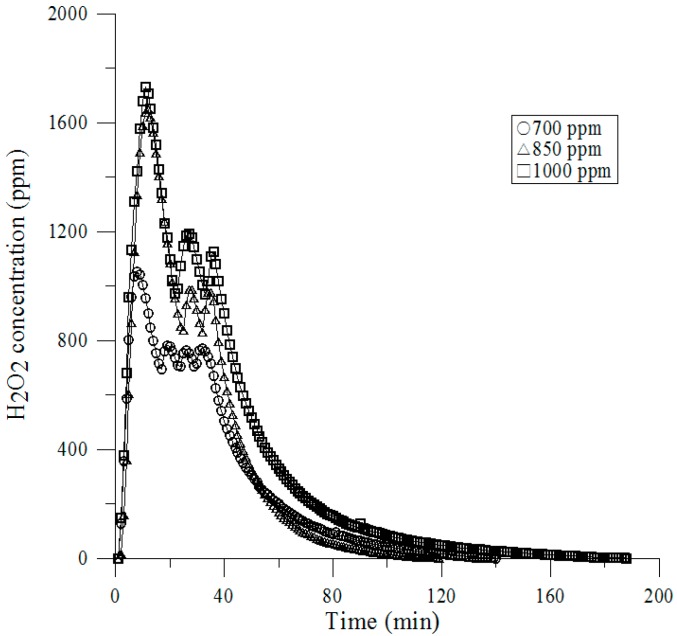
Survivors vs. VPHP (Vaporized Hydrogen Peroxide) Concentration.

We conducted the determination of vaporized H_2_O_2_ of a sufficient concentration to complete the decontamination. No conditioning phase was used and the H_2_O_2_ was vaporized and injected over a period of only a few minutes, as shown in Figure 7. After completing the injection phase, the dwell periods of various lengths were tested and operated during the recirculation blower of the RTP. Initially the RTP was dehumidified to 5% RH and various injection concentrations were tested. A rather sharp break point can be observed at 1054, 1068, and 1730 ppm at 700, 850, and 1000 ppm, respectively. The injection rate of 700 ppm resulted in one to three minutes. Table 3 present the results of those that were extremely reproducible and representative experiments.

The performance of the RTP is important, especially in ensuring the minimization of cross-contamination of the RTP integrity and HEPA filter efficiency. The RTPs are normally tested for airflow velocity, particle counts, pressure decay of leakage, and sterility. All of these parameters must be set at pre-defined ranges.

It has been found that the air flow balance is affected by the quantity of airflow and the height above the platform of the RTP. It is relatively easy to evaluate the RTP leak with pressure decay, where the system is appointed with the air, closed, and the decay of pressure is measured via time. We conducted the determination of vaporized H_2_O_2_ of a sufficient concentration to complete the decontamination compliance with acceptance testing procedure of FDA guideline [27] continuously.

## 4. Conclusions

The performance of the RTP chamber is important, especially in ensuring the minimization of contamination of the RTP chamber integrity and the HEPA filter efficiency. The RTP chambers are normally tested for airflow velocity, particle counts, pressure decay of leakage, and sterility. All of these parameters must be set at pre-defined ranges. It has been found that the air flow balance of the RTP chamber is affected by the quantity of the airflow and the height above the platform. It is relatively easy to evaluate the RTP chamber leak with pressure decay, where the system is ordered via the air, closed, and the sink of pressure is measured versus time. We conducted the determination of vaporized H_2_O_2_ of a sufficient concentration to complete the decontamination. This study details a procedure for validation of the RTP chamber. The approach to performance testing described here is generally applicable; however, the results are directly applicable to only the product tested. Performance testing of the RTP chamber will improve safety and can be completed at an ISO Class 5 environment.

## Figures and Tables

**Table 1:** Measured concentrations of particles of various sizes at the measuring points given in Figure 3.

**20 cm over Platform, 75% Airflow Quantity**					
Measuring points	A1	B1	A2	B2	M
0.3 µm	456	6634	1427	301	956
0.5 µm	33	475	96	26	76
1 µm	9	158	38	4	14
3 µm	0	12	0	0	1
5 µm	1	7	2	0	1
10 µm	0	0	0	0	0
**20 cm over Platform, 60% Airflow Quantity**					
Measuring points	A1	B1	A2	B2	M
0.3 µm	436	3035	2673	533	393
0.5 µm	29	197	178	37	30
1 µm	8	76	66	10	6
3 µm	1	9	3	2	2
5 µm	0	2	1	0	1
10 µm	0	0	0	0	1
**20 cm over Platform, 50% Airflow Quantity**					
Measuring points	A1	B1	A2	B2	M
0.3 µm	1165	11,781	937	802	520
0.5 µm	76	854	59	54	34
1 µm	19	294	20	18	8
3 µm	0	23	0	1	0
5 µm	0	9	0	0	0
10 µm	0	0	0	0	0
**40 cm over Platform, 75% Airflow Quantity**					
Measuring points	A1	B1	A2	B2	M
0.3 µm	88	30,766	27,221	2488	0
0.5 µm	5	2387	2103	205	0
1 µm	3	764	705	46	0
3 µm	0	42	50	0	0
5 µm	0	20	24	2	0
10 µm	0	1	0	0	0
**40 cm over Platform, 60% Airflow Quantity**					
Measuring points	A1	B1	A2	B2	M
0.3 µm	1772	38,530	78,428	1712	0
0.5 µm	130	2941	5473	115	0
1 µm	38	1050	1665	30	0
3 µm	6	100	145	3	0
5 µm	2	50	75	2	0
10 µm	0	2	3	1	0
**40 cm over Platform, 50% Airflow Quantity**					
Measuring points	A1	B1	A2	B2	M
0.3 µm	7642	7937	124,631	665	84
0.5 µm	530	575	8763	50	10
1 µm	148	172	2566	16	2
3 µm	11	7	195	1	0
5 µm	2	12	74	0	0
10 µm	0	0	5	0	0
**60 cm over Platform, 75% Airflow Quantity**					
Measuring points	A1	B1	A2	B2	M
0.3 µm	45,210	3492	4218	5322	1
0.5 µm	3432	265	295	414	0
1 µm	1092	84	88	96	0
3 µm	74	5	7	9	0
5 µm	42	3	2	3	1
10 µm	3	1	0	1	0
**60 cm over Platform, 60% Airflow Quantity**					
Measuring points	A1	B1	A2	B2	M
0.3 µm	1971	33,288	18,861	43,865	1
0.5 µm	159	2513	1376	3361	0
1 µm	42	851	370	1076	0
3 µm	4	57	26	76	0
5 µm	1	32	13	36	0
10 µm	0	1	1	2	0
**60 cm over Platform, 50% Airflow Quantity**					
Measuring points	A1	B1	A2	B2	M
0.3 µm	2034	89,337	15,599	4054	1
0.5 µm	120	6895	1123	262	0
1 µm	40	2174	341	79	0
3 µm	6	172	24	3	0
5 µm	2	82	10	2	0
10 µm	0	2	2	0	0

**Table 2:** Correlations of pressure (*P*) vs. time (*t*).

*P_i_* (Pa)	Pressure (*P*) vs. Time (*t*)	*P_f_* (Pa)
45	*P* = −0.0033 ln(*t*) + 4.0742, *R*^2^ = 0.9612	7.2
30	*P* = −0.0036 ln(*t*) + 3.5563, *R*^2^ = 0.961	5.6
15	*P* = −0.0242 ln(*t*) + 3.3191, *R*^2^ = 0.9775	2.2

**Table 3:** Survivors vs. Total Concentration of H_2_O_2_.

Total Concentration of Sterilant	700 ppm	850 ppm	1000 ppm
Positive BIs/Total BIs	0/120	120/120	0/120
